# Granular cell tumor of the breast: a case report and review of literature

**DOI:** 10.4076/1757-1626-2-8551

**Published:** 2009-08-10

**Authors:** Vanja Filipovski, Saso Banev, Vesna Janevska, Blagica Dukova

**Affiliations:** Institute of Pathology, Medical Faculty, University of “St Cyrill and Methodius”Street Vodnjanska bb, Skopje, 1000Republic of Macedonia

## Abstract

A 22-year-old female patient presented with a breast mass lesion with a clinical suspicion of a fibroadenoma. Histological evaluation revealed a rare benign neoplasm - granular cell tumor.

Granular cell tumor is rare neoplasm that may arise in virtually any body site, and in 5% it occurs in the breast. The histogenesis of this tumor is still rather controversial and currently the most acceptable theory is a Schwann cell origin. The main histological feature is granular cytoplasm of the tumor cells.

From a clinical point of view there is a similarity between granular cell tumor and mammary carcinoma on mammography and ultrasound. Pathohistologically, sometimes, differential diagnostic difficulties exist concerning apocrine carcinoma, histiocytic lesions and metastatic neoplasms.

## Introduction

The first report of granular cell tumor is attributed to Abrikossoff in 1926 localized in the tongue and he proposed origin from striated muscle cells and he termed this lesion as myoblastoma. Tissue culture studies of three granular cell tumors appeared to support this interpretation. Subsequently, accumulated evidence has cast doubt on this theory. Some authors showed evidence of a histiocytic derivation, while others proposed a possible origin from smooth muscle cells. The most widely accepted theory has been that of a Schwann cell origin, apparently because of the positivity of the tumor for the S-100 protein [1] and the similarities exhibited in the ultrastructural features of the tumor cells and those of Schwann cells [2].

The granular appearance of the cytoplasm may be caused by accumulation of secretory granules, mitochondria, or lysosomes. Mital et al. [3] showed evidence of granule origin as infoldings of cell membrane by a process similar to myelin formation around nerves. They further argued that the subsequent phagocytosis of the infoldings by lysosomes results in the characteristic cytoplasmic granules.

This tumor may present differential diagnostic difficulty since it may simulate carcinoma on mammography and ultrasound.

## Case presentation

A surgeon examined a 22-year-old Caucasian female college student, complaining of a breast lump. Ultrasound and mammography revealed a well-defined breast mass suspicious of fibroadenoma. The patient had no prior operations and no pregnancies, and there was not any family history of breast tumor. The operative material was received in our institute with the clinical diagnosis of fibroadenoma. The operation and the postoperative period were uneventful. To date there are no recurrences of the lesion.

On gross examination the material was composed of eight soft tissue fragments measuring from 1 to 2.5 centimeters in diameter. Parts of the fragments were gray-white solid tumor masses and the other fragments were recognized as breast tissue.

**Figure 1.**
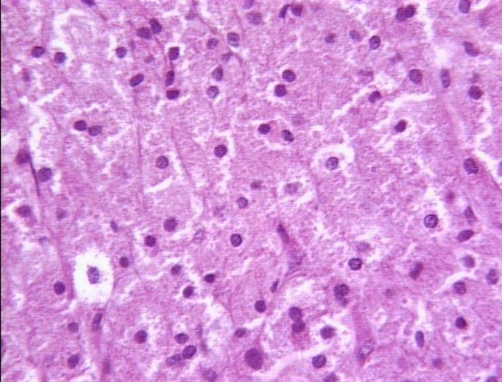
Hematoxylin-Eosin × 400: Typical eosinophilic granular appearance of the cytoplasm.

**Figure 2.**
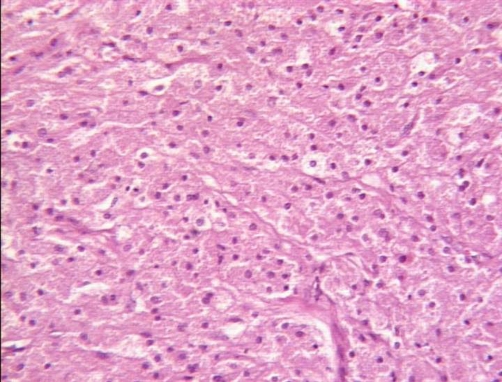
Hematoxylin-Eosin × 200: Spindle to polygonal cells arranged in compact nests and sheets.

**Figure 3.**
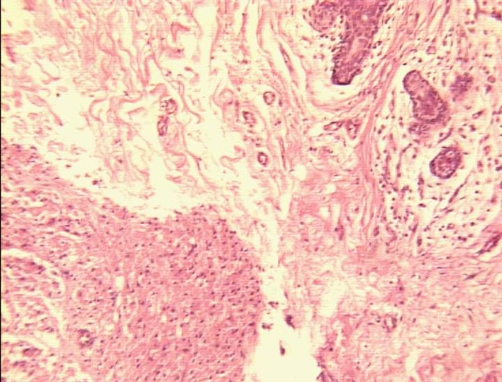
Hematoxylin-Eosin × 100: Peripheral area of the where the tumor (left) shows a well defined pushing border. On the right top corner breast lobular units are evident.

**Figure 4.**
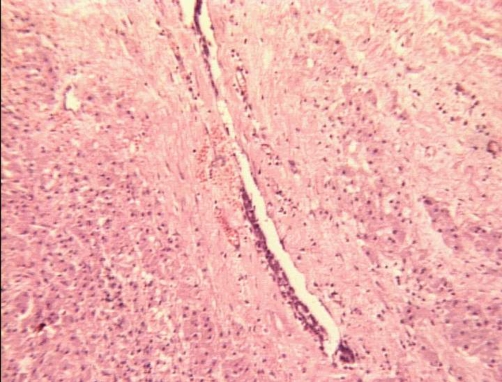
Hematoxylin-Eosin × 100: Peripheral area of the where the tumor (left) shows infiltrative growth (right) around a breast ductal structure (center).

**Figure 5.**
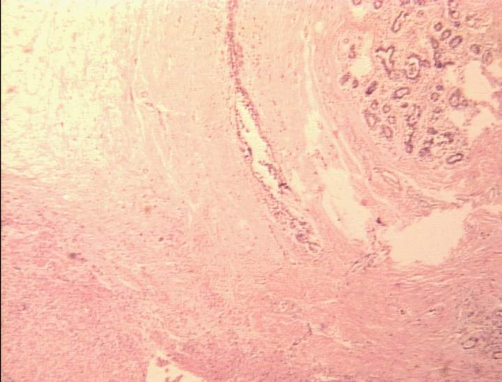
Hematoxylin-Eosin × 40: Panoramic view of the lesion. The tumor is in the lower left corner, fat lobules on the upper right corner, ductal unit in the top center portion, and a breast lobular unit on the upper right corner.

Microscopically a benign neoplastic tumor was observed. The tumor was composed of compact nests and sheets of cells with well-defined cell borders that contain eosinophilic cytoplasm granules. The cells were polygonal to spindle in shape. Nuclei are round to slightly oval and some of them contain prominent nucleoli (Figure 1,2). In several focuses especially on the periphery of the lesion a prominent lymphoplasmocytic infiltration is observed. Rarely in the periphery, nerve bundles were seen. Mostly the tumor was well defined, however, focuses of infiltrative growth existed (Figure 3,4). Around the lesion normal breast tissue persisted (Figure 5).

**Figure 6.**
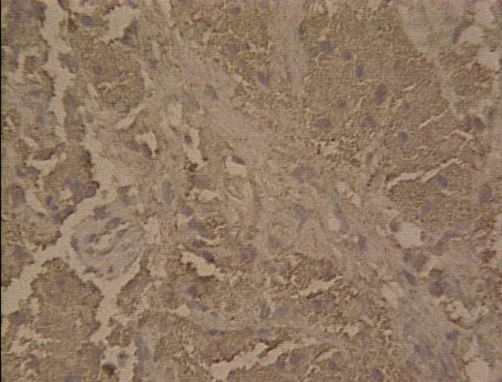
S-100 protein × 200: positive tumor cells.

The immunohistochemical analysis showed the following results: Positive staining for S-100 protein (Figure 6), vimentin, carcinoembryonic antigen, CD68, neuron specific enolase, and negative staining for cytokeratin proteins, MAC387, glial fibrilar acidic protein actin, desmin, and synaptophysin.

## Discussion

Granular cell tumors arise throughout the body while these lesions in the breast account for 5% of all cases [4,5]. These tumors most commonly occur in women between 30 and 50 years of age ranged from 17 to 74 years with a frequency approximately 1 in 1000 breast cancers [6]. Some authors show ethnic propensity in women of African American origin [5]. The tumor arises from the intralobular stroma of the breast. Although virtually all the lesions are benign neoplasms, rare malignant lesions are described [7].

In most cases there is a firm hard mass in any part of the breast, but more commonly in the upper inner quadrant of the breast, which differs from carcinomas that arise more commonly in the upper outer quadrant [2], and this distribution appear to correspond to the area of innervation of the skin of the breast by the supraclavicular nerve [4]. Usually the tumor appears as a solitary unilateral lesion, but rarely multiple lesions in the breast and other parts of the body are seen.

On mammography granular cell tumor of the breast is difficult to distinguish from carcinoma [8], forming typical stellate mass lacking calcifications with the dense core [9]. Ultrasound usually reveals a solid mass with posterior shadowing suggestive of carcinoma [9]. Rarely the ultrasound pattern is hypoechoic with or without attenuation of the sound beam [10].

Granular cell tumor usually presents as a firm or hard mass. Most of the tumors appear to be well circumscribed. Other examples have ill-defined infiltrative borders. The cut surface is white or gray, or it may have a yellow-two-tan color. Lesions measuring up to 6 cm have been reported, but the tumors generally are 3 cm or smaller.

With very rare exceptions granular cell tumor of the breast is a benign neoplasm. The tumor is indistinguishable from those of granular cell tumors arising at other sites. The lesion is composed of compact nests or sheets of cells that contain eosinophilic cytoplasmic granules. The granules usually are prominent and fill the cytoplasm with the tendency of cytoplasmic vacuolization and clearing. The cytoplasmic granules are diastase resistant and PAS positive. Cell borders typically are well defined, and the cells vary in shape from polygonal to spindle. Variable amounts of collagenous stroma are present. Nuclei are round to slightly oval with an open chromatin pattern, and nucleoli tend to be prominent [11]. In some cases a modest amount of nuclear polymorphism and occasional multinucleated cells may be found but these features should not be interpreted as evidence of malignant neoplasm. Small nerve bundles sometimes are seen in the tumor or in close association in the periphery of the lesion [12].

Granular cell tumor should be distinguished from mammary carcinoma, histiocytic lesions and metastatic neoplasms. The infiltrative character of the tumor with cells containing prominent nucleoli, especially when the lesion has collagenous stroma closely resemblance scirrhous carcinoma. Strong, diffuse immunoreactivity for S-100 protein and carcinoembryonic antigen that is typical for granular cell tumor may also be seen in certain cancers. A high proportion of granular cell tumors are reactive for vimentin, which is detectable in relatively few carcinomas, and granular cell tumor is negative for estrogen and progesterone receptors. This lesion may be similar to apocrine carcinoma. The presence of intraductal carcinoma often with lobular extension, as well as cytological polymorphism, usually serves to identify apocrine carcinoma. Apocrine carcinomas are immunoreactive for cytokeratin and usually for epithelial membrane antigen, while granular cell tumor is not positive for epithelial markers and does not contain mucin. The difference between granular cell tumor and granulomatous inflammatory reaction or a histiocytic tumor is negativity for histiocyte-associated antigens, but reactivity for CD68 has been described in granular cell tumor [13] as in our case. Granular cell tumor must be distinguished from metastatic neoplasm in the breast that have oncocytic or clear cell features, such as renal carcinoma, malignant melanoma and alveolar soft part sarcoma [8].

Granular cell tumor of the breast is treated by wide excision. Local recurrence may occur after incomplete excision, but sometimes it is difficult to distinguish between recurrence and asynchronous multifocal lesions. Direct invasion of an axillary lymph node by a granular cell tumor of the breast that arose in the axillary tail has been reported [10]. Less than 1% of all granular cell tumors, including mammary lesions are malignant. Systemic metastases have been described in patients with non-mammary malignant granular cell tumors [14]. One patient presented with a 4 cm breast tumor and multiple pulmonary metastases that were confirmed histological to be metastatic granular cell tumor [15]. Another patient was found to have axillary metastasis when a tumor initially excised from the upper anterior chest wall recurred in her right breast [7].

## Conclusion

Granular cell tumor is a rare benign neoplasm of uncertain origin. Only 5% of these tumors occur in the breast. Clinically using mammography and ultrasound, these tumors can often simulate more ominous lesions like carcinomas. The main striking histological element is the presence of granular cytoplasm, and these tumors should be distinguished from carcinomas with oncocytic appearance, histiocytic lesions and metastatic carcinomas.

**Figure 7.**
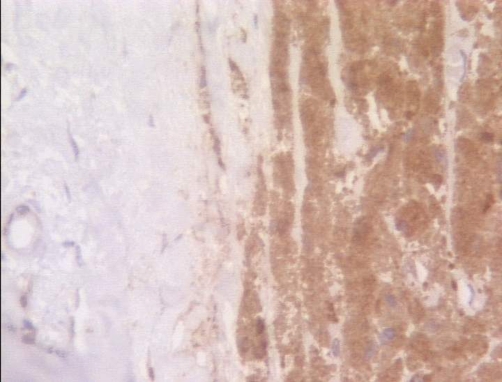
CD-68 × 200: positive tumor cells on the right, negative staining of breast parenchyma on the left.

## Abbreviations

CD: Cluster of differentiation
MAC: Macrophage monoclonal antibody
PAS: Periodic_acid-Schiff_stain
